# Phenotype-Genotype analysis of caucasian patients with high risk of osteoarthritis

**DOI:** 10.3389/fgene.2022.922658

**Published:** 2022-08-29

**Authors:** Yanfei Wang, Jacqueline Chyr, Pora Kim, Weiling Zhao, Xiaobo Zhou

**Affiliations:** Center for Computational Systems Medicine, School of Biomedical Informatics, The University of Texas Health Science Center at Houston, Houston, TX, United States

**Keywords:** knee osteoarthritis, genome-wide association study, genetic correlation, comorbidities, mendelian randomization

## Abstract

**Background:** Osteoarthritis (OA) is a common cause of disability and pain around the world. Epidemiologic studies of family history have revealed evidence of genetic influence on OA. Although many efforts have been devoted to exploring genetic biomarkers, the mechanism behind this complex disease remains unclear. The identified genetic risk variants only explain a small proportion of the disease phenotype. Traditional genome-wide association study (GWAS) focuses on radiographic evidence of OA and excludes sex chromosome information in the analysis. However, gender differences in OA are multifactorial, with a higher frequency in women, indicating that the chromosome X plays an essential role in OA pathology. Furthermore, the prevalence of comorbidities among patients with OA is high, indicating multiple diseases share a similar genetic susceptibility to OA.

**Methods:** In this study, we performed GWAS of OA and OA-associated key comorbidities on 3366 OA patient data obtained from the Osteoarthritis Initiative (OAI). We performed Mendelian randomization to identify the possible causal relationship between OA and OA-related clinical features.

**Results:** One significant OA-associated locus rs2305570 was identified through sex-specific genome-wide association. By calculating the LD score, we found OA is positively correlated with heart disease and stroke. A strong genetic correlation was observed between knee OA and inflammatory disease, including eczema, multiple sclerosis, and Crohn’s disease. Our study also found that knee alignment is one of the major risk factors in OA development, and we surprisingly found knee pain is not a causative factor of OA, although it was the most common symptom of OA.

**Conclusion:** We investigated several significant positive/negative genetic correlations between OA and common chronic diseases, suggesting substantial genetic overlaps between OA and these traits. The sex-specific association analysis supports the critical role of chromosome X in OA development in females.

## Introduction

As one of the top reasons for morbidity and disability, osteoarthritis (OA) has affected over 32.5 million adults in the United States (Barbour et al., 2017). In 2004, the cost of OA management was $336 billion, or 3% of the United States’ GDP. As the most common form of arthritis, the economic burden of OA is quickly growing due to obesity prevalence and aging. By 2030, an estimated 20 percent of Americans (about 70 million people) will be at risk of developing this disease (Chu et al., 2012; Yelin et al., 2016). OA is a disease of the whole joint based on the characterized degenerative changes in bone, cartilages, menisci, and ligaments (Zhang et al., 2016; Chen et al., 2017; Zhang et al., 2019). The subsequent pain and stiffness usually cause inconveniences in patients’ daily lives. Unfortunately, OA is still an irreversible degenerative disease without effective disease-specific drugs (Sharma et al., 2001a; Boer et al., 2021), and current treatment focuses on ameliorating symptoms such as pain relief. The pathology of OA disease is still unclear. Thus, there is an urgent need to understand disease etiopathology and identify new drug targets.

Twin studies and family-based epidemiologic studies have revealed the heritability of OA susceptibility (Palotie et al., 1989; Spector et al., 1996; Felson et al., 1998). One sibling study estimated the heritability for knee OA at 0.62 (Neame et al., 2004), suggesting a substantial involvement of genetic factors in the development of the OA. Many efforts have been devoted to identifying the genetic biomarkers for the diagnosis of OA, where they used Kellgren-Lawrence (KL) grade (KL ≥ 2) to define OA occurrence. Several candidate genes have been identified to be associated with the diagnosis of knee OA such as GDF5, NCOA3, CHST11, FTO, and ALDH1A2 (Valdes et al., 2011; Loughlin, 2015; Yau et al., 2017; Takuwa et al., 2018). Zengini et al. reported nine genetic loci over the genome-wide significance threshold in GWAS (Zengini et al., 2018). Rs3815148 on chromosome 7 is found to influence susceptibility for the prevalence of OA (Kerkhof et al., 2010; Evangelou et al., 2011).

Most OA-related studies have focused on the influence of genetic factors on OA, and few studies have explored the genetic links between OA and other chronic diseases. Like other aging diseases, the prevalence of comorbidities among patients with OA is high. A recent epidemiological study found that patients with OA have twice as many comorbid conditions as those without OA. (Marshall et al., 2019). A meta-analysis of 42 studies reports that thirty-five percent of OA patients have cardiovascular disease, sixteen percent have ulcers and 14 percent have diabetes (Louati et al., 2015; Swain et al., 2020). Although the dominant approach of GWAS is to identify the association between one single-nucleotide polymorphism (SNP) and a binary disease indicator, multiple studies have revealed some diseases may be genetically correlated (Piva et al., 2015; Courties and Sellam, 2016; Barowsky et al., 2021; Zhang et al., 2022). In another word, these diseases may share the same genetic variants. Therefore, we conducted a phenotype-genotype comorbidity analysis of patients with a high risk of OA using the Osteoarthritis Initiative (OAI) data. We also performed multi-genotype tests to detect OA genetic variations related to OA clinical phenotypes. Despite the higher prevalence of OA in women, traditional GWAS often excludes sex chromosomes, leading to the unknown role of the chromosome X in OA (Laitner et al., 2021). Therefore, we performed sex-specific GWAS to fill this gap.

## Materials and methods

### Study design and analysis plan

To identify the potential genetic biomarkers associated with OA in Caucasians, genome-wide associations were performed on the OAI dataset using logistic regression in PLINK (Chang et al., 2015). In this cohort, participants aging between 45 and 79 years old were recruited from five different clinical sites, including the University of Maryland School of Medicine, the Ohio State University, the University of Pittsburgh, Memorial Hospital of Rhode Island, and the University of California (https://nda.nih.gov/oai). Longitudinal MRI/CT images, genotyping data, and clinical information was analyzed to evaluate potential biomarkers and characterize OA incidents and progression (Peterfy et al., 2008; Eckstein et al., 2012; Eckstein et al., 2014). The assessed GWAS results were adjusted for age, gender, and BMI. The results with a *p*-value $p\leq 10^{-5}$ and linkage disequilibrium value *r* ^2^ < 0.6 were selected for the subsequent analysis. (Marchini et al., 2007). The summary statistics were then used in the further genetic correction analysis and Mendelian randomization (MR) analysis. We also used metaCCA (Cichonska et al., 2016) for data reduction for genotype and phenotype separately with multiple univariate GWAS results and then calculated the correlation between genotype-phenotype association using canonical correlation analysis. The workflow for sample selection and study design was described in Supplementary Figure S1.

### Phenotype definition and study populations

**FIGURE 1:**
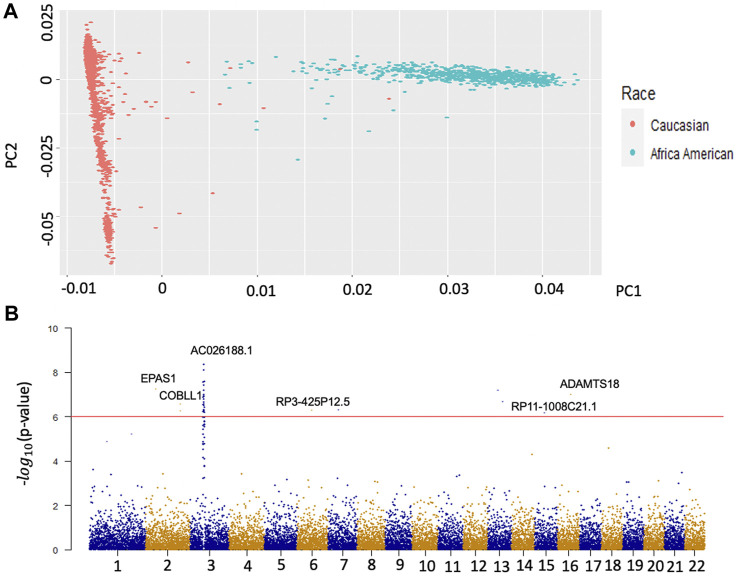
Population Stratification of two main races and Manhattan plots of GWAS for OA. Panel A is the result of the first two principal components. The red dots represent the population structure of Caucasians on the whole genotype matrix. The green dots represent the population structure of African Americans on the whole genotype matrix. Panel B is the Manhattan plot of the results from the GWAS of Caucasian OA. The y axis represents –log (*p* values) for the association of variants with OA. The horizontal red line represents the threshold for genome-wide significance.

Population stratification is a primary confounder in GWAS, causing false-positive signals. Therefore, we performed principal component analysis (PCA) on two major races, Caucasian and African American, to identify the population structure with common variants. The structure of genetic variants of the two populations is significantly different in Figure 1A. A total number of 3366 OA Caucasian patients were selected from the OAI database. 1,498 patients are males and 1868 are females. To identify the genetic variants associated with OA, we conducted a GWAS based on this dataset. The OA disease was defined as KL score ≥ 2 (Kellgren and Lawrence, 1957). KL ranges from 0 to 4, with 0 indicating no sign of OA and 4 representing severe OA. After filtering and quality control, 1872 cases and 1,316 controls were retained for further analysis. The control group was defined as patients without any sign of OA and the experimental group was defined as OA patients whose KL score was ≥2. In addition to OA, comorbidity disease survey data, including asthma, heart disease, stroke, diabetes, and cancer, were also extracted. 281 out of 3297 OA patients also had asthma (ICD-9 code 493.92). 99 out of 3316 OA patients reported prior incidence of stroke (ICD-9 code 434.91). 62 out of 3,300 OA patients reported heart problems (ICD-9 code 429.9). 189 out of 3,307 OA patients reported having Type I or Type II diabetes (ICD-9 code 250.00). 130 out of 3,316 OA patients reported having cancer (ICD-9 code 199.1).

In addition to these binary disease indicators, continuous OA-related phenotypes such as The Western Ontario and McMaster Universities Osteoarthritis (WOMAC) pain score, WOMAC stiffness, knee alignment, the total area of subchondral bone, and medial bone mineral density (BMD) were included. These participants were the same group of available KL score population. The most common symptoms of OA are pain and stiffness. WOMAC pain score, ranging from 0 to 20, uses five questionnaires to self-assess pain levels with activities of daily living. WOMAC stiffness, similar to WOMAC pain score, ranges from 0 to 8. Knee alignment (hip-knee-ankle alignment) and BMI are known as risk factors for the incidence and progression of OA with different joint loading measurements (Messier et al., 2014). The pathology of OA is characterized by modeling subchondral bone (Donell, 2019). The total area of subchondral bone and medial BMD are both indicators for the formation of osteophytes (Xiao et al., 2010; Donell, 2019).

### Genotyping and imputation

OAI participants were genotyped with Illumina Omni-Quad 2.5 M arrays, including genotype information for 2,440,283 SNPs. SNPs with low minor allele frequency (MAF <1%) were excluded. Genotype imputation was conducted using IMPUTE2 (Browning and Browning, 2016) with 1000 Genomes Project Phase 3 as the reference and 8,248,570 SNPs in OAI were obtained.

### Genetic correlation analysis

For phenotype-genotype comorbidity analysis, the complex trait analysis (GCTA) tool (Yang et al., 2011) was used to assess the variance explained by all the SNPs on the whole genome. To identify common diseases that share genetic architecture with OA, linkage disequilibrium (LD) score regression from LD Hub was used (Zheng et al., 2017). The additive genetic covariance between two traits was scaled by the square root of the product of each trait’s genetic variance to determine the genetic correlation (Ni et al., 2018). Instead of using individual-level genotype data, the genetic correlation $r_{g}$ is estimated from GWAS summary statistics by calculating the LD score for each SNP. When an SNP is able to tag more of its neighbors, the LD score for this SNP is higher, which is more likely to affect the phenotypes (Lee et al., 2018). The slope of LD Score regression estimates the heritability $\left(h^{2}\right)$ of this trail. We downloaded 856 published GWAS clinical trials on LD Hub (Zheng et al., 2017) and the trials whose genetic correlation with *p* values <0.05 were considered significant with Bonferroni correction for multiple testing.

### Mendelian randomization

To identify the role of exposure (knee alignment/pain) in the susceptibility of OA, two-sample Mendelian randomization (MR) analysis was performed on the MR-Base platform (Walker et al., 2019). MR uses significantly associated SNPs as instrumental variables to quantify causal relationships between risk factors and OA. By including genetic variants, MR reduced the impact of confounding, even “reverse causality” based on Mendel’s second law (Bennett and Holmes, 2017). Specifically, the causal relationship is estimated by computing the association between identified significant SNPs for the risk factor and OA. Figure 5A illustrates the estimation of MR. The causal associations were estimated by three different methods, namely, inverse variance weighted (IVW), MR–Egger (Bowden et al., 2015), and weighted median (WM). The IVW method uses the Wald ratios to estimate the causal effects of each SNP and is easily biased by inverse variance or pleiotropic effects. MR–Egger is a weighted linear regression under the assumption that pleiotropic associations are independent. The WM is useful when at least 50% of effects came from inverse variances. In general, we performed GWAS on pain, and knee alignment using the OAI dataset respectively and obtained SNPs that were strongly associated ( $p\leq 10^{-5}$ ) and independent inheritance (*r* ^2^ < 0.6). Finally, we extracted the instrumental SNPs from the KL GWAS. For linkage disequilibrium, we used clumping to prune SNPs.

### Gene-set and tissue expression analysis by FUMA

FUMA (Watanabe et al., 2017) uses GTEx (Ardlie et al., 2015) data for tissue-specific expression patterns and identifies tissue specificity of prioritized genes. Based on the GWAS summary statistics, FUMA identifies significant independent SNPs and genomic loci based on LD structure. The significant independent SNPs are then annotated on gene function using ANNOVAR (Wang et al., 2010) based on Ensembl and their effects on gene expression using eQTLs of various tissue types. The enrichment of significant genes and functional categories is evaluated by a hypergeometric test.

## Results

### Identification of genetic loci associated with OA by genome-wide association study

**FIGURE 2:**
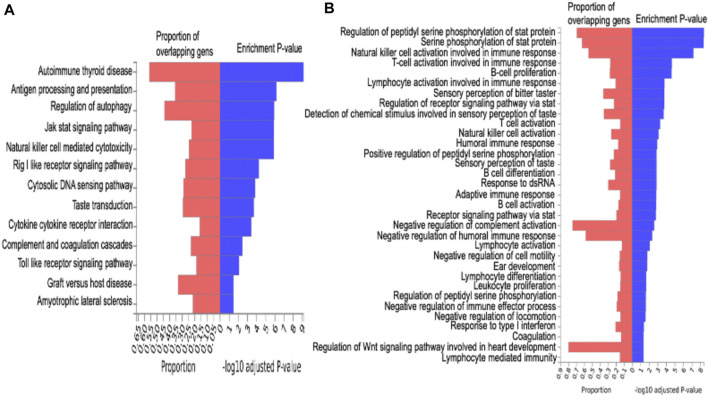
Enrichment analysis. Figure A is the KEGG pathway analysis for functional enrichment clustering analysis. Figure B is gene ontology (GO) enrichment analysis.

To eliminate unknown population-associated sub-structures, this study focused on the Caucasian population. The OA GWAS result is presented in Figure 1B. We identified 164 significant variants mapped on 79 genes ( $p\leq 10^{-5}$ ) with PLINK and used the online database Enrichr (Chen et al., 2013) to conduct a pathway analysis and Gene Ontology (GO) terms enrichment. A total of 116 genes were significantly enriched in 13 KEGG pathways and 33 GO terms (Figure 2). In each pathway, the minimum number of genes was 3. The pathways with a Bonferroni correction test P<0.05 are considered significant. Autoimmune thyroid disease is the most significant pathway with $p\leq 10^{-9}$ . By mapping genes to GO terms, we found that most GO terms were related to phosphorylation and immune response. One significant SNP candidate was found on chromosome 2 with multiple loci overlapping with EPAS1 (rs6707241, $p=9.33*10^{-7}$ ) (Supplementary Figure S2A). EPAS1 (also called HIF2α) has been known as a hypoxia regulator (Saito et al., 2010). Bone-related study indicates that HIF2α is a regulator of osteoblastogenesis and bone mass accrual (Merceron et al., 2019). Hence, HIF-2alpha may represent a therapeutic target for osteoarthritis. Another interesting SNP was within the ADAMTS18 gene (rs12443792, $p=9.82*10^{-7}$ ) (Supplementary Figure S2B). Some members of the ADAMTS family are expressed in cartilage and have emerging roles in joint pathophysiology (Yang et al., 2017).

### Local genetic correlation between OA and common comorbidities

**FIGURE 3:**
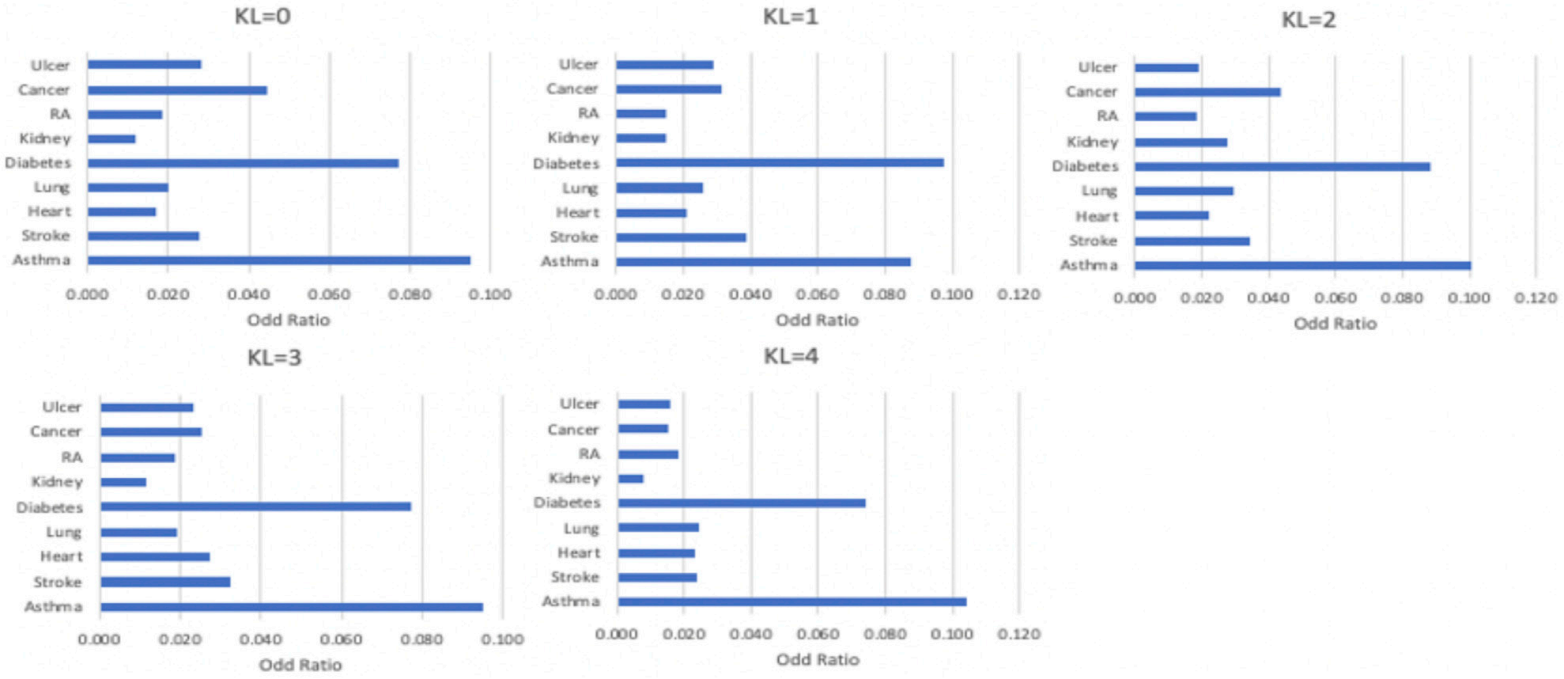
The distribution of comorbidity in different OA severity.

As shown in Figure 3, comorbidities are significantly associated with OA severity. For example, strokes were more likely to be found in patients with KL = 1, patients with KL = 2 are more likely to have diabetes and asthma, and heart diseases mostly occurred in patients with KL = 3. To identify the common loci of significance to most comorbidities, we performed the GWAS on different comorbidity diseases. Based on these GWAS results, we found significant positive correlations between OA and heart disease (coefficient = 0.5, se = 0.66) and stroke (coefficient = 0.2, se = 0.88). Table 1 listed the correlation between each significant SNP and common OA comorbidities. The first column is the overall estimated correlation for each single loci in risk factors. The second column is the *p*-value, while the significant threshold is set as $\frac{0.05}{2440283}=2.05*10^{-8}$ . The third and following columns are the coefficients corresponding to that phenotype. The largest single-SNP–multi-traits $-log_{10}\left(p-value\right)$ are 10.5164 for KIAA1211 (chr4-57072329), 9.75 for SLC4A4 (chr4-72371592), and 10.08 for RP11-333A23.4 (chr8-71393927). Functional assays showed that solute carrier family 4 members 4 (SLC4A4) might serve as a potent Rheumatoid Arthritis-specific target (Torres et al., 2022). KIAA1211, also known as Cancer-related Regulator of Actin Dynamics (or CRAD), was found to regulate cell proliferation and apoptosis in non-small cell lung cancer (NSCLC) *in vitro* and *in vivo* (Liu et al., 2019). While the gene function of RP11-333A23.4 (lincRNA) and KIAA1211 are documented in other diseases, their functions in OA pathways are still unknown.

**TABLE 1:** The lead SNPs at independent loci reaching $p\leq 10^{-8}$ at multi-trails.

SNP	rg	$-log_{10}\left(p\right)$	KL	Stiffness	Pain	BMI	Alignment
SNP4-57072329	0.129	10.164	−0.226	1.474	−1.851	−0.055	0.072
SNP4-57033235	0.129	10.163	−0.226	1.474	−1.851	−0.054	0.072
SNP8-71393927	0.128	10.077	−0.579	1.688	−0.932	−0.172	−0.270
SNP7-4485777	0.127	9.842	0.020	1.511	−1.123	−0.768	-0.140
SNP4-72371592	0.127	9.749	−0.212	−0.992	1.495	0.571	0.333
SNP7-4482664	0.125	9.505	−0.039	−1.480	1.104	0.792	0.148
rs4500015	0.117	8.127	−0.340	1.873	−1.215	−0.224	−0.146

### Global genetic correlation between osteoarthritis and other complex traits

**FIGURE 4:**
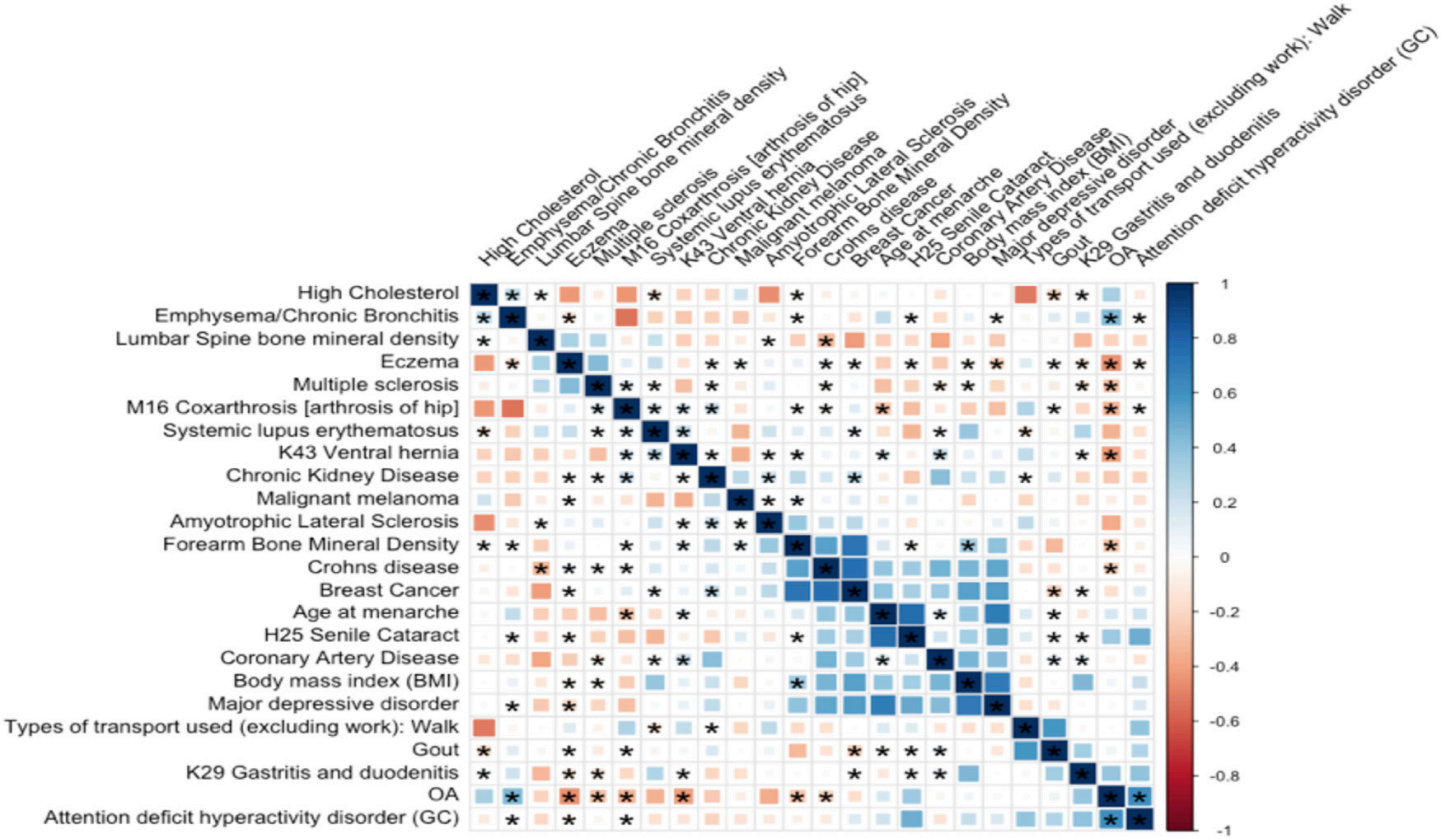
Genetic correlation between OA and 23 highly correlated disease traits. The square color corresponds to $r_{g}$ . A positive genetic association is shown in blue, whereas a negative genetic correlation is represented by red. The *p*-value is proportional to the size of the colored squares, with larger squares representing a lower *p*-value. Asterisks indicate genetic associations that differ from 0 at P < 0.05.

The correlation matrix between OA and a subset of common diseases is represented in Figure 4. Our analysis highlights eight significant genetic correlations for knee OA (Figure 4). The negative genetic correlation between knee OA and M16 coxarthrosis/arthrosis of the hip ( $r_{g}=-0.32$ , s.e. = 0.22, *p* = 0.033) are consistent with previous epidemiological and genetic studies (Ro et al., 2019; Liu et al., 2020). We also identified some novel genetic correlation results, which have not been reported in the GWAS study. First, we found a positive genetic correlation between knee OA and emphysema/chronic bronchitis ( $r_{g}=0.34$ , s.e. = 0.18, *p* = 0.0537), which was consistent with epidemiological results (Melville et al., 2010; Kim and Criner, 2013; Wshah et al., 2018). Second, the estimate of a negative genetic correlation between knee OA and forearm bone mineral density ( $r_{g}=-0.31$ , s.e. = 0.20, *p* = 0.0934) suggested that the same genetic factors influenced normal variation in bone mineral density (BMD) regardless of OA patients or normal people. This result agreed with the observation that BMD implicated epigenetic marks (Stewart and Black, 2000; Hochberg et al., 2004; Chan et al., 2014; Hardcastle et al., 2015). Third, we found a positive genetic correlation between attention deficit hyperactivity disorder (ADHD) and OA ( $r_{g}=0.214$ , s.e. = 0.118, *p* = 0.057). In a recent study, ADHD was found to be associated with 14 autoimmune diseases, including Crohn’s disease, diabetes, multiple sclerosis, and rheumatoid arthritis (Li et al., 2019). We also estimated a negative genetic correlation between multiple sclerosis (MS) and OA ( $r_{g}=-0.24$ , s.e. = 0.11, *p* = 0.129), which was consistent with epidemiological evidence (Tseng et al., 2016). The comorbidity of OA has not been fully studied in autoimmune diseases and may raise the possibility of similarity between these autoimmune diseases. Last, the estimate of a negative genetic correlation between OA and Crohn’s disease ( $r_{g}=-0.228$ , s.e. = 0.19, *p* = 0.035) is consistent with arthritis as a presenting symptom of Crohn’s disease (Ergül et al., 2012). The positive genetic correlation between age at menarche and OA is consistent with previous epidemiological reports (Hellevik et al., 2017) although it is not statistically significant. We also identified two unpublicized associations which required further analysis. First, we estimated a statistically significant negative genetic correlation between OA and eczema ( $r_{g}=-0.325$ , s.e. = 0.19, *p* = 0.0654), suggesting a further investigation. Second, we estimated a negative genetic correlation between k43 ventral hernia and OA ( $r_{g}=-0.427$ , s.e. = 0.24, *p* = 0.54).

### Causal relationship between known clinical indicators and osteoarthritis

**FIGURE 5:**
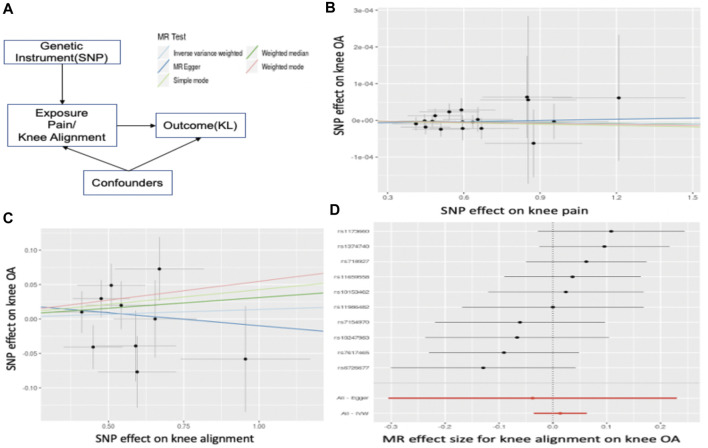
Mendelian Randomization. **(A)** illustrates the estimation of MR. In **(B),** the x-axis represents the effect of SNPs on pain level and the y-axis is the effect of SNPs on OA. The slope is the estimated causal effect. In **(C),** the x-axis represents the effect of SNPs on abnormal knee alignment levels and the y-axis is the effect of SNPs on OA. **(D)** listed significant SNPs linking knee alignment level and OA. Each dot represents a SNP, and the line represents the 95% confidence interval.

Pain and abnormal knee alignment are common symptoms of OA. Figure 5A shows the estimated causal associations of clinical indicators (pain, knee alignment) with OA based on Mendelian randomization (MR). The effects of SNP on pain and OA are shown in Figure 5B. There is no causal relationship between pain and risk of knee OA since the slopes of all MR lines were close to 0. In Figure 5C, 4 MR methods, including IVW, simple mode, weighted mode, and weighted median, identified a role for knee alignment in OA risk. Having alignment of more than 5 degrees (in either direction) in both knees at baseline was associated with significantly greater functional deterioration during the 18 months than having alignment of 5 degrees or less in both knees (Sharma et al., 2001b). Figure 5D listed significant SNPs linking knee alignment level and OA.

### Sex-specific genes at identified loci

**FIGURE 6:**
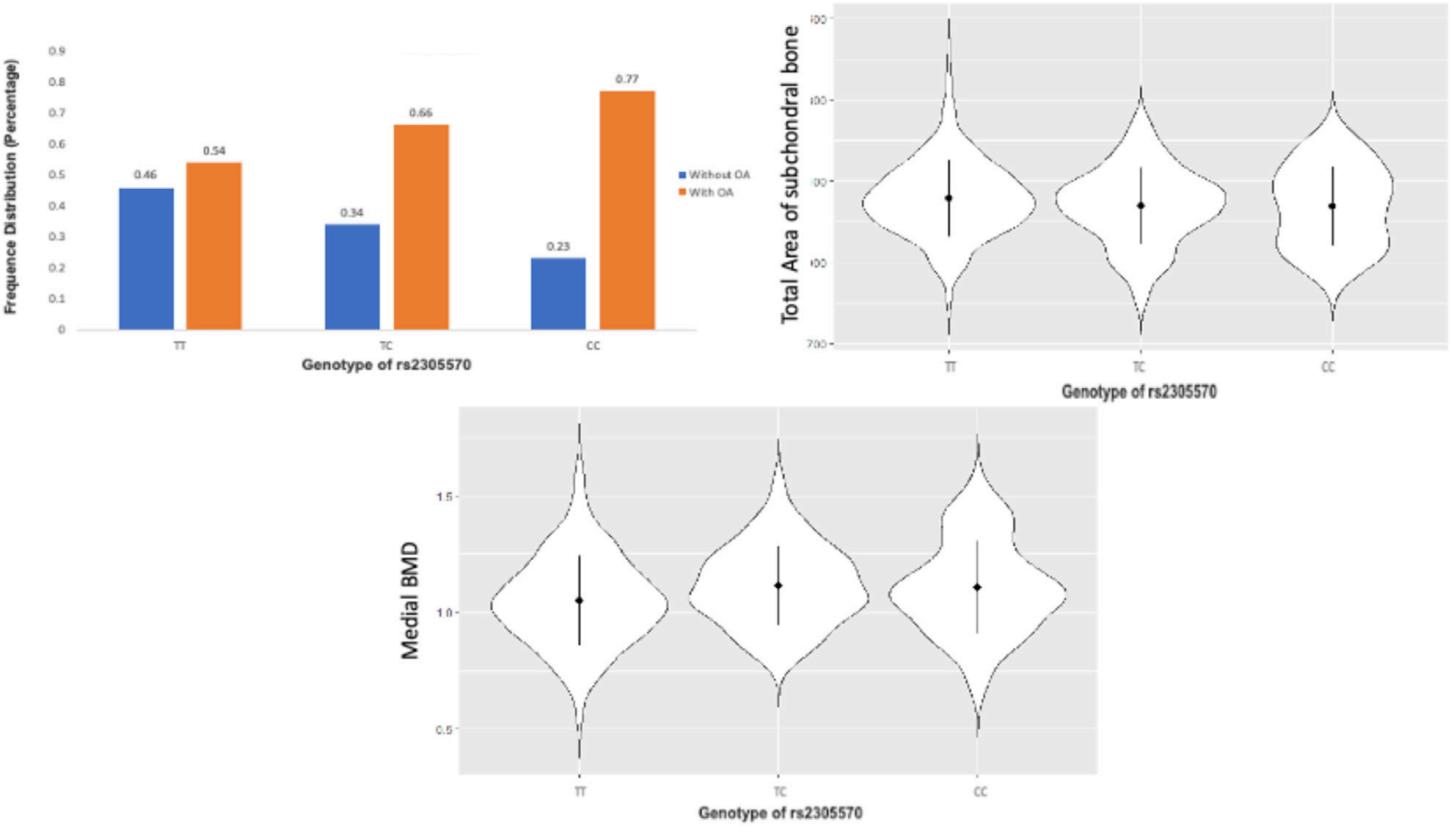
The TT, TC, and CC genotypes of rs2305570 had a different performance on the total area of subchondral bone, and medial BMD.

We conducted an association analysis to estimate the contribution of the chromosome X to OA generic variance. One significant locus rs2305570 was identified to be significantly associated with OA. The different genotypes of this SNP also perform differently on the total area of subchondral bone, and medial bone mineral density (BMD), indicating this locus may be related to the bone area and medial BMD (Figure 6). SNP rs2305570 is in the coding region of KIAA1210 (ENST00000402510.2) and introduces a non-synonymous mutation. SNPs that occur in protein-coding regions, especially non-synonymous SNPs (nsSNPs), can change the amino acid encoding the mutation site and may result in the amino acid variants in proteins (Yates and Sternberg, 2013), leading to structural and functional changes in protein structures. An important paralog of KIAA1210 is SPOCK1 (SPARC/Osteonectin). SPOCK1 is a positive downstream regulator of transforming growth factor-β (TGF-β) (Sun et al., 2020). The TGF-β signaling pathway is critical for maintaining homeostasis in OA-affected joints (van der Kraan, 2018). Based on protein-protein interactions (PPI), TXLNG is highly associated with KIAA1210. TXLNG is found with the ability to regulate bone mass accrual in mouse protein from UniProtKB by inhibiting activating transcription factor 4-mediated transcription.

## Discussion

Studies have shown that women are more likely to have OA than men (Hame and Alexander, 2013; Tschon et al., 2021; Srikanth et al., 2005; O'Connor, 2006). In general, among people over 60 years old, approximately 10% of men and 13% of women have symptomatic knee OA (Zhang et al., 2010). We identified a novel susceptibility locus at rs2305570 located within the exons of the KIAA1210 gene. KIAA1210 is crucial in regulating bone mass density through an ATF4-dependent pathway. Alteration in KIAA1210 is found to be associated with maintaining homeostasis in joints affected by osteoarthritis.

We also noticed age at menarche was negatively correlated with OA. In other words, the increasing age at menarche reduced the risk of OA. This is also in line with previous clinical trials in which women who started menstruating before age 11 had a 9–15% higher risk of knee replacement surgery as they got older (Bayliss et al., 2017). This may link androgens to knee OA and explain why women are more likely to have OA than men. Therefore, hormone therapy may improve symptoms of knee OA.

We found heart disease and stroke were the two most significant comorbidities genetically related to OA. A previous meta-analysis confirmed a strong (Wang et al., 2016) genetic correlation between OA and heart disease. Obesity is a major contributor to OA and heart disease. Obesity puts extra stress on both the joints and heart, which can cause damage over time. Aging is the major contributor to both OA and stroke. In addition, we found several significant positive and negative genetic correlations between OA and 4 common diseases (emphysema/chronic bronchitis, Crohn’s disease, eczema, and arthrosis of hip), showing that these features may play a role in the genetic etiology of OA. Aside from that, we discovered that knee OA was genetically positively correlated to a long smoking history as it was tied to lifestyle and environment.

We tested whether structural changes and clinical indicators have causal effects on OA. Our results indicate that pain and sometimes stiffness are not causal factors for OA, but abnormal knee alignment is a significant risk factor. By considering the multiple phenotypes together, this method increased statistical power for some SNPs than the univariate tests separately (Cichonska et al., 2016). This joint analysis of the genetic and imaging/clinical features provides an opportunity to uncover the genetic variants of OA development, including genetic factors that are related to OA mechanism. As a result, we have identified seven significant loci significantly associated with OA and its common symptoms (See Table 1).

Although OA is not considered a traditional inflammatory disease, both pathway analysis and GO enrichment terms are shown that immune responses are tightly related to OA. A significant correlation is detected between OA and Crohn’s disease (see Figure 4), which is a common disease of inflammation. These results are generally consistent that OA does involve inflammation. There are several limitations to our study. We excluded rare SNPs with allele frequencies below 1%. This limited our power to detect lower-frequency variants. In addition, this study focused on the genetic correlation between OA and its comorbidities in Caucasians and the results may not be applicable for other sub-populations. Additional samples will be needed for further meta-analysis. Meanwhile, due to genetic confounding, the interpretation of genetic correlation and MR were challenging. For example, in Figure 4, OA was statistically genetically related to multiple inflammation diseases. This could result from a direct causal effect of inflammation → OA could also be caused by an unknown confounder such as inflammation ← G →OA, where G is the set of genetic variants with effects on both inflammation and OA. In summary, we performed GWAS for radiographic knee OA, its comorbidities, and common clinical measurements. We also investigated the genetic correlations and causal effects. Furthermore, we also extended our association analysis of OA to the chromosome X.

## Data Availability

The genotype data used in this article is from this dbGaP Study Accession: phs000955.v1.p1 (Genetic Components of Knee Osteoarthritis (GeCKO) Study: The Osteoarthritis Initiative). Below is the link to this study: https://www.ncbi.nlm.nih.gov/projects/gap/cgi-bin/study.cgi?study_id=phs000955.v1.p1.

## Supplementary material

Supplementary Figure S1 Study design.

Supplementary Figure S2 LocusZoom for significant signals for GWAS results (– log10 (*p* value)) for genotyped SNPs are plotted against genomic position. The circles indicate association results of genotyped SNPs in the region; the color reflects the correlation coefficient (r2) of each genotyped SNP in 1000 Genomes Project of November 2014 Eur. Estimated recombination rates (in cM/Mb) are plotted in blue. The region shown in the plots extends to either 500 kb upstream or downstream of the index SNP.
