# Supplementary figures and images for: Phenotype-Genotype analysis of caucasian patients with high risk of osteoarthritis
Source: Front Genet. 2022 Aug 29;13:922658. doi: 10.3389/fgene.2022.922658 (PMC9465622; doi:10.3389/fgene.2022.922658)

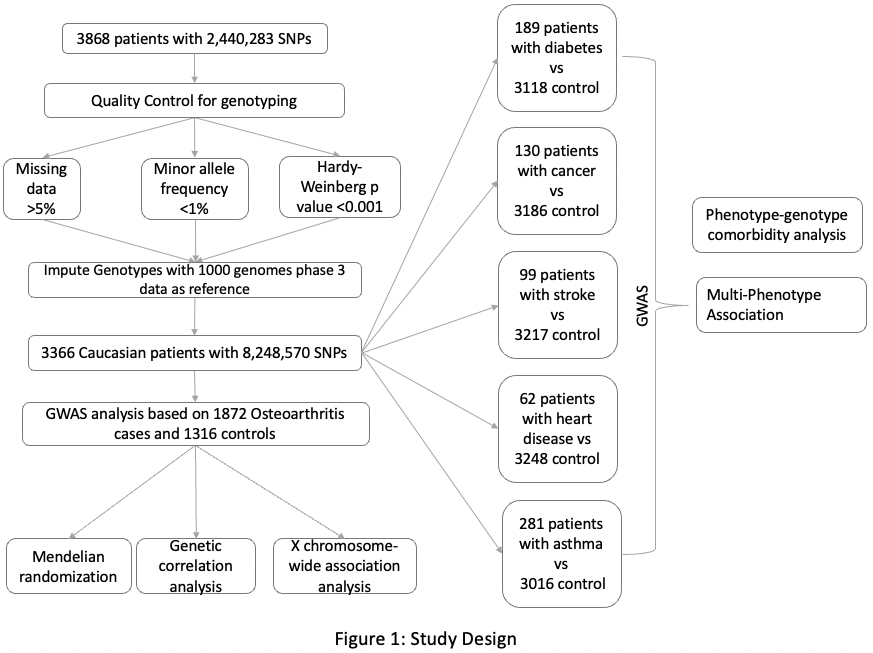

Supplement: Supplementary file 1 [file Image1.tiff]

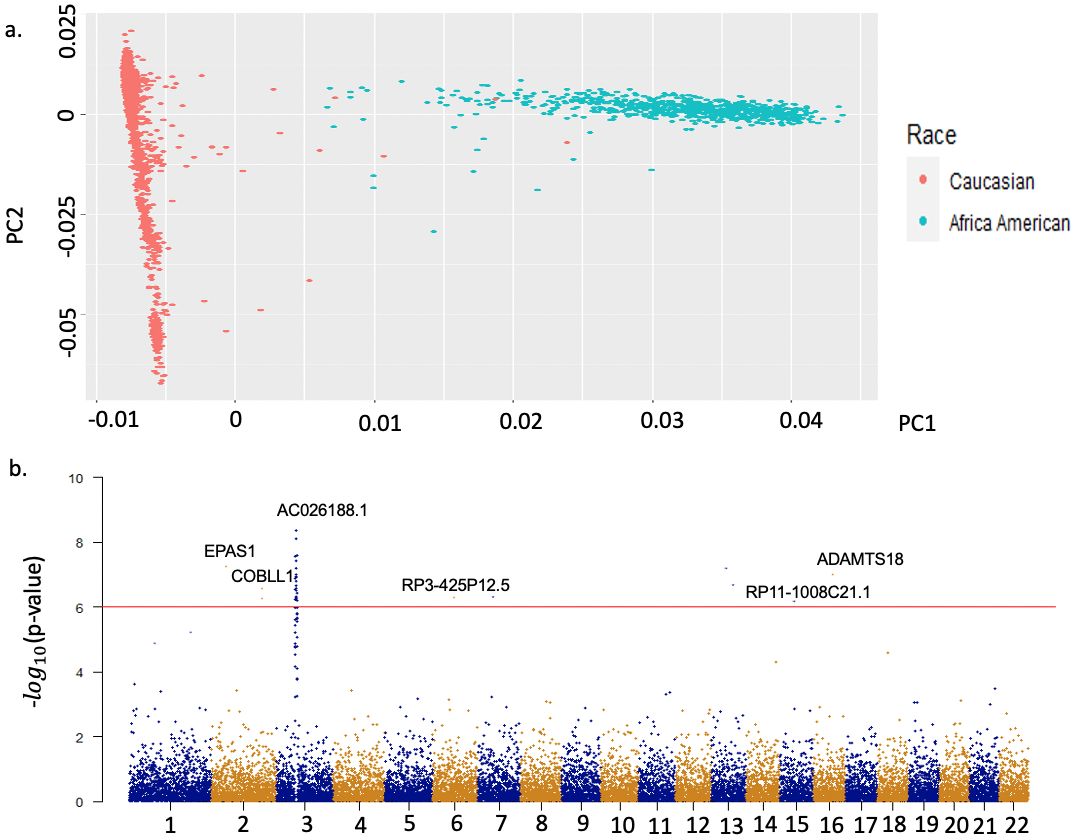

Supplement: Supplementary file 3 [file Image2.tiff]
